# Comparative genome analysis of *Sesbania cannabina*-nodulating *Rhizobium* spp. revealing the symbiotic and transferrable characteristics of symbiosis plasmids

**DOI:** 10.1099/mgen.0.001004

**Published:** 2023-05-03

**Authors:** Kunming Han, Yan Li, Zhenpeng Zhang, Liqin Sun, En Tao Wang, Yan Li

**Affiliations:** ^1^​ Yantai Key Laboratory of Characteristic Agricultural Bioresource Conservation & Germplasm Innovative Utilization, College of Life Sciences, Yantai University, Yantai, Shandong 264005, PR China; ^2^​ Key Laboratory of Coastal Biology and Bioresource Utilization, Yantai Institute of Coastal Zone Research, Chinese Academy of Sciences, Yantai, PR China; ^3^​ Departamento de Microbiología, Escuela Nacional de Ciencias Biológicas, Instituto Politécnico Nacional, Mexico City 11340, Mexico

**Keywords:** conjugal transfer, *Rhizobium*, secretion system, *Sesbania cannabina*, symbiotic plasmid

## Abstract

Symbiotic nitrogen fixation between legumes and rhizobia makes a great contribution to the terrestrial ecosystem. The successful symbiosis between the partners mainly depends on the *nod* and *nif* genes in rhizobia, while the specific symbiosis is mainly determined by the structure of Nod factors and the corresponding secretion systems (type III secretion system; T3SS), etc. These symbiosis genes are usually located on symbiotic plasmids or a chromosomal symbiotic island, both could be transferred interspecies. In our previous studies, *Sesbania cannabina*-nodulating rhizobia across the world were classified into 16 species of four genera and all the strains, especially those of *Rhizobium* spp., harboured extraordinarily highly conserved symbiosis genes, suggesting that horizontal transfer of symbiosis genes might have happened among them. In order to learn the genomic basis of diversification of rhizobia under the selection of host specificity, we performed this study to compare the complete genome sequences of four *Rhizobium* strains associated with *S. cannabina*, YTUBH007, YTUZZ027, YTUHZ044 and YTUHZ045. Their complete genomes were sequenced and assembled at the replicon level. Each strain represents a different species according to the average nucleotide identity (ANI) values calculated using the whole-genome sequences; furthermore, except for YTUBH007, which was classified as *Rhizobium binae *, the remaining three strains were identified as new candidate species. A single symbiotic plasmid sized 345–402 kb containing complete *nod*, *nif*, *fix*, T3SS and conjugal transfer genes was detected in each strain. The high ANI and amino acid identity (AAI) values, as well as the close phylogenetic relationships among the entire symbiotic plasmid sequences, indicate that they have the same origin and the entire plasmid has been transferred among different *Rhizobium* species. These results indicate that *S. cannabina* stringently selects a certain symbiosis gene background of the rhizobia for nodulation, which might have forced the symbiosis genes to transfer from some introduced rhizobia to the related native or local-condition-adapted bacteria. The existence of almost complete conjugal transfer related elements, but not the gene *virD*, indicated that the self-transfer of the symbiotic plasmid in these rhizobial strains may be realized via a *virD*-independent pathway or through another unidentified gene. This study provides insight for the better understanding of high-frequency symbiotic plasmid transfer, host-specific nodulation and the host shift for rhizobia.

## Data Summary

The genome sequences of the four *Rhizobium* strains YTUBH007, YTUZZ027, YTUHZ044 and YTUHZ045 sequenced in this study were deposited in the National Center for Biotechnology Information database under the accession numbers PRJNA856439, PRJNA843512, PRJNA856444 and PRJNA856733, respectively.

**Impact Statement**

*Sesbania cannabina*-nodulating rhizobia were classified into 16 species of four genera but with highly conserved symbiotic genes, especially for *Rhizobium* spp., indicating they acquired the genes through horizontal transfer. To uncover the transfer mechanism of these genes, the complete genome sequences of four *Rhizobium* strains were obtained and analysed. We revealed the rhizobia acquired the symbiotic plasmid through a conjugal transfer process but using a *virD*-independent pathway. In addition, we found the symbiotic plasmid of *Agrobacterium pusense* IRBG74 could not be transferred due to the absence of necessary conjugal transfer elements. This study provides insight for the better understanding of rhizobial symbiotic plasmid transfer and evolution.

## Introduction

Rhizobia are Gram-negative bacteria known for their ability to elicit root or stem nodules on legume hosts to establish nitrogen-fixing symbiosis. This kind of symbiosis is important to the terrestrial ecosystem for contributing half of global terrestrial nitrogen compounds [1]. The nodulation process is elicited through the rhizobial synthesis of Nod factors after detection of the flavonoid compounds secreted by the legume host, then the Nod factors induce root hair curling, formation of the infection thread and division of the cortical cells. Finally, the rhizobial cells enter the cortical cells and differentiate to nitrogen-fixing bacteroids [2]. In this mutualism association, the legume host benefits from absorbing ammonium compounds synthesized by rhizobia, while the bacteria receive other nutrients, mainly carbon sources, produced by the host [3].

The nodulation process involves a highly complex molecule exchange interaction, in which the products of *nod*, *nif* and secretion-system-related genes play key roles [3]. These genes are usually located on a large symbiotic plasmid, the so called symbiotic plasmid (pSym), in fast-growing *Rhizobium* and *Sinorhizobium* species, and on the symbiotic island in slow growing *Bradyrhizobium* species and some species of *Mesorhizobium* [4–6]. Both pSym or symbiotic island could be transferred interspecies or even inter-genera through conjugal transfer [4, 6, 7], so called horizontal gene transfer (HGT), resulting in a symbiotic phenotype shift. The successful self-transfer of a plasmid needs some essential elements: an *oriT* site and a conjugative transfer system. The *oriT* region is usually tens to hundreds of base pairs in length, contains a conserved nick region (flanking the *nic* site) and variable numbers of inverted repeats [8]. The *nic* site can be recognized and cleaved by a relaxase; however, the inverted repeats are involved in the localization to a precise *nic* site as well as the termination of ssDNA transfer [8, 9]. The conjugative transfer system encompasses two parts: the transfer (*tra*) genes involved in DNA transfer and replication (Dtr), and the mating pair formation (Mpf) component [10]. The Dtr set includes proteins participating in DNA relaxation (*traA*), pilus formation and ssDNA transfer (*traCDG*) [11, 12], while the Mpf is composed of *trb* or *virD4* (type IV secretion system; T4SS) related genes [10, 13, 14]. However, the expression of both Dtr and Mpf genes is regulated by TraI, CinI, TraR and CinR [15]. The quorum-sensing regulation is generated by the product of *cinI* or *traI*, which produces l-homoserine lactone to form complexes with TraI or CinI regulators, then the transcription of Dtr and Mpf genes involved in conjugation is induced [15, 16].

As a legume species belonging to the tribe *Sesbanieae* of subfamily *Papilionoideae*, *Sesbania cannabina* is an annual semi-shrub native to the South Pacific Islands and has spread in Asia, Africa and Europe (https://www.iucnredlist.org/species/168726/20141760). It forms nitrogen-fixing root nodules with rhizobia belonging to 16 species in four genera [17]. The rhizobia associating with *S. cannabina* showed biogeographical patterns: the *Rhizobium* species were mainly distributed in acid environments; whereas the *Sinorhizobium* species were dominant in alkaline saline conditions [17, 18]. Interestingly, all the *Sesbania*-nodulating rhizobia harboured highly conserved symbiosis genes and formed a mono clade in the phylogenetic tree of these genes, indicating HGT events [17]. Among the *S. cannabina-*nodulating rhizobia, the strains of *Rhizobium* spp. contained extraordinarily conserved symbiosis genes, indicating the possibility that they may have acquire these genes recently.

Previously, events of horizontal transfer of symbiosis genes have been estimated among the rhizobia nodulating with chickpea [19], common bean [20, 21], soybean [22] and some other plants grown in China. Comparative genomic studies on genes inside and outside the symbiosis islands have revealed the possibility that successful HGT needs compatibility between the transferred genes and the genomic background [22, 23]. In this study, genomes of four *S. cannabina-*nodulating *Rhizobium* strains were sequenced by combining the PacBio and Illumina HiSeq platforms, and were assembled at the replicon level; the genome characteristics of the pSyms were analysed. The identities, phylogenetic relationships, symbiotic properties and the transferrable abilities among these pSyms were characterized and compared. We aimed to determine the genomic features and evolution for the transferrable symbiotic plasmids.

## Methods

### Rhizobial strains

In this study, four representative strains for *S. cannabina-*nodulating rhizobia isolated from weakly acidic–neutral soils (pH 5.92–7.34), including *Rhizobium binae* YTUBH007, *Rhizobium* sp. YTUHZ045, *Rhizobium* sp. YTUHZ044 and *Rhizobium* sp. YTUZZ027 [17], were used for a comparative genomic study with two other genome sequences of *S. cannabina-*nodulating strains *Sinorhizobium alkalisoli* YIC4027^T^ [24] and *Agrobacterium pusense* IRBG74 [25], which were download from GenBank. Almost identical *nodA* gene sequences (98.8–100 % similarities) were detected among the *Rhizobium* and *Sinorhizobium* strains, and the *nodA* gene of the *A. pusense* strain shared similarities of 92.2–92.4 % with those of the other five strains [17]. These strains formed a sample set with similar symbiosis genes, but different species–genus backgrounds.

### Genome sequencing, assembly and annotation

Genomic DNA was extracted separately from strains *R. binae* YTUBH007, *Rhizobium* sp. YTUZZ027, *Rhizobium* sp. YTUHZ044 and *Rhizobium* sp. YTUHZ045 by using a genome extraction kit for bacteria (Sangon Biotech), according to the manufacturer’s instructions. The DNA samples were examined by a NanoDrop spectrophotometer (Thermo) and then fragmented for genomic DNA library preparation. Then, the Illumina PE library (400 bp) and PacBio library (20 kb) were constructed and sequenced with the Illumina HiSeq 2500 platform with 150 bp paired-end technology and the PacBio RSII platform, respectively, at Biozeron Biotechnology.

The raw reads were checked using FastQC and then pruned/filtered by Trimmomatic (v0.38) [26] to obtain clean reads. The clean Illumina reads were assembled by using SOAPdenovo (v2.04) [27], and blasR [28] was used to compare the PacBio reads, then the Celera Assembler 8.0 [29] was selected to connect scaffolds to obtain the complete genome sequences. The complete genome sequences were annotated by using Prokka (v1.14.6) [30] and eggNOG (v2.1.4) [31]. The genome sequences of strains *Sinorhizobium alkalisoli* YIC4027^T^ [18] and *A. pusense* IRBG74 were extracted from the GenBank database for comparative study. The genome characteristics were counted for all the six tested strains by using Python (v3.9.12). The plasmids with nodulation genes *nodABC* and iron-nitrogenase-encoding gene *nifH* were classified as pSyms in this study.

### Comparison of the complete genome and plasmid sequences

For this comparison, the genome sequences of several reference strains representing other rhizobia were extracted from the GenBank database, and were analysed together with those acquired in this study. The core genes among either the complete genome or pSyms were analysed by Orthofinder (v2.5.4) [32]. A maximum-likelihood phylogenetic tree was reconstructed based on the concatenated single copy core sequences by using iq-tree 2.2 [33] and then visualized by iTOL (https://itol.embl.de/). Average nucleotide identity (ANI) values between the pair of complete genomes or pSym sequences were calculated by using the OrthoANI program [34], and the amino acid identity (AAI) values were calculated by CompareM (v0.1.2) (https://github.com/dparks1134/CompareM). The heatmaps were generated according to the ANI and AAI values by using R (v4.1.0).

### Gene annotation for pSyms

eggNOG (v2.1.4) [31] was used to annotate the pSym sequences. The results were assessed based on Clusters of Orthologous Groups (COGs) using ‘stringr’ and ‘dplyr’ packages from the R software. The Kyoto Encyclopedia of Genes and Genomes (KEGG) annotation process for pSyms was performed by using the online program (https://www.genome.jp/kegg/mapper/reconstruct.html).

### Analyses of symbiosis, nitrogen fixation and conjugal transfer related genes

The protein sequence database [including *nod*, *nif*, *fix*, type III secretion system (T3SS) and conjugative transfer related genes] was constructed according to previous studies [35]. The alignment for each pSym was performed by using each reference sequence in the database using blast software version 2.13.0. The genetic organization plots for these genes were drawn using the online program ‘gene cluster' (https://xiaochi.chifei3d.com/static/xiaochiPlot/src/gene_cluster.html). The *oriT* for each pSym was predicted using the online oriTfinder prediction program [36].

## Results

### Genomic characteristics of *S. cannabina*-nodulating *Rhizobium* spp.

**Fig. 1.**
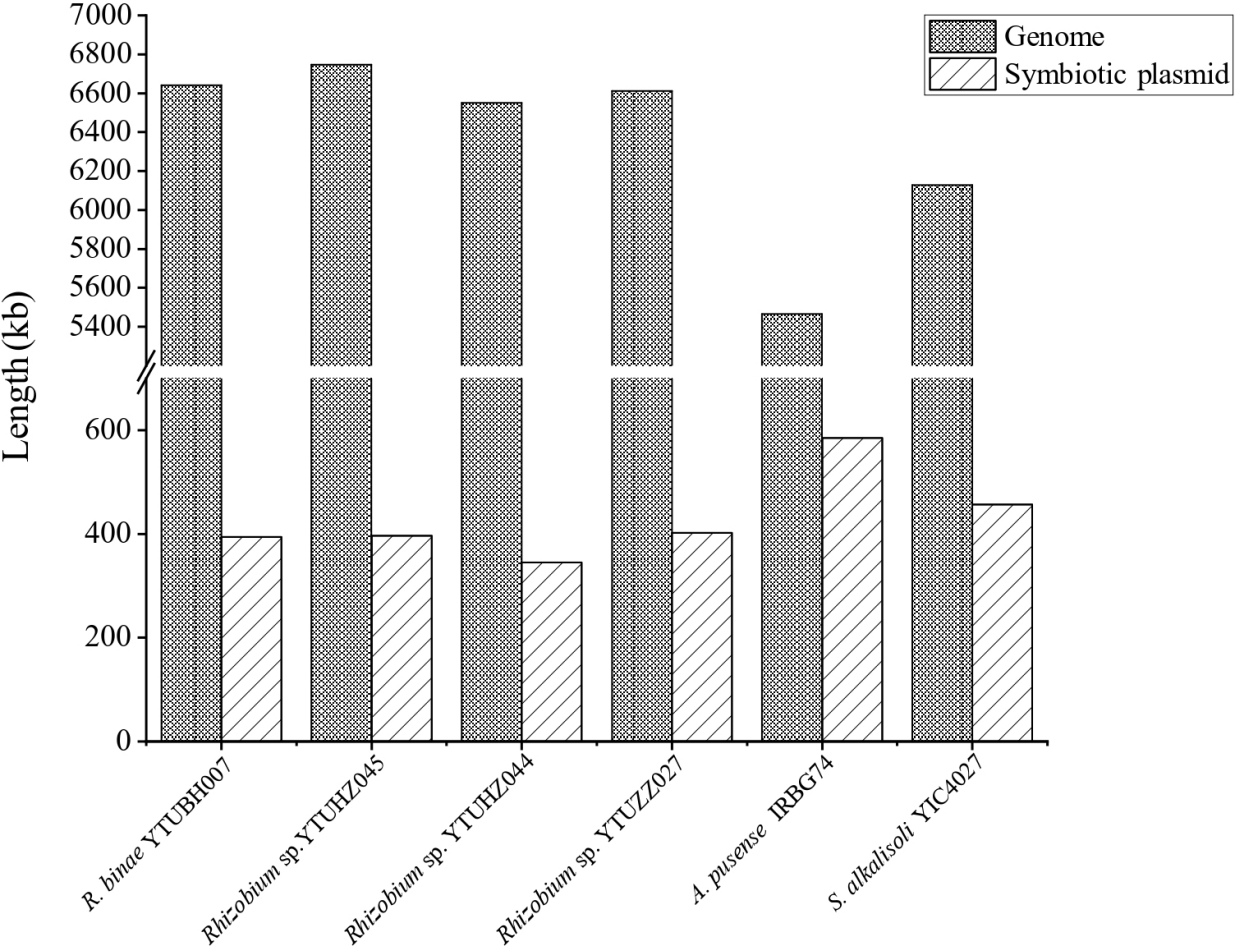
The complete genome and symbiotic plasmid sizes of *S. cannabina*-nodulating rhizobia.

The complete genome sequences of the four tested *Rhizobium* strains obtained in this study were at the replicon level, with genome sizes of 6.55 to 6.75 Mb and 3–7 plasmids (Figs 1 and S1, Table S1, available with the online version of this article). The symbiotic plasmid sizes range from 344 to 402 kb and the G+C content is 58.43–59.09 mol%, which is obviously lower than for the complete genome sequences (Figs 1 and S1, Table S1). The gene functions of pSym coding sequences (CDSs) detected in all the six tested strains were classified into COGs families composed of 19 categories (Table S2) and three main function classes (>10 % of the total CDSs): replication, recombination and repair (L); energy production and conversion (C); amino acid transport and metabolism (E); which accounted for 20.57, 10 and 10 % of the total classified CDSs, respectively. Meanwhile, 17 % of the CDSs were classified as function unknown (S). In addition, the CDSs of the pSyms were classified into 27 KEGG pathways (Table S3), with the major pathways of membrane transport, amino acid metabolism, carbohydrate metabolism and xenobiotics biodegradation and metabolism, representing 17, 16, 15 and 15 % of total CDSs, respectively. These prediction results indicate that these rhizobia harbour many genes corresponding to the transportation of amino acids and carbohydrate and energy metabolism, which are related to the function of the high energy consuming nitrogen fixation process and the nutrient exchange between rhizobia and the host plant in nodule symbiosis.

### Phylogenetic and homologue analyses of the whole genomes and pSyms

**Fig. 2.**
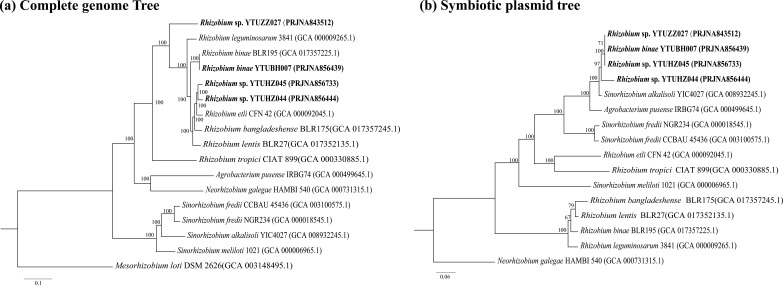
Maximum-likelihood phylogenetic tree based on the whole-genome sequences (**a**) and the symbiotic plasmid (pSym) sequences (**b**). The trees were reconstructed using iq-tree based upon the common shared single copy orthologue genes of the complete genome (**a**) and the pSyms (**b**). Bootstrap support values were calculated from 1000 replicates，scale bar units are number of substitutions per site.

**Fig. 3.**
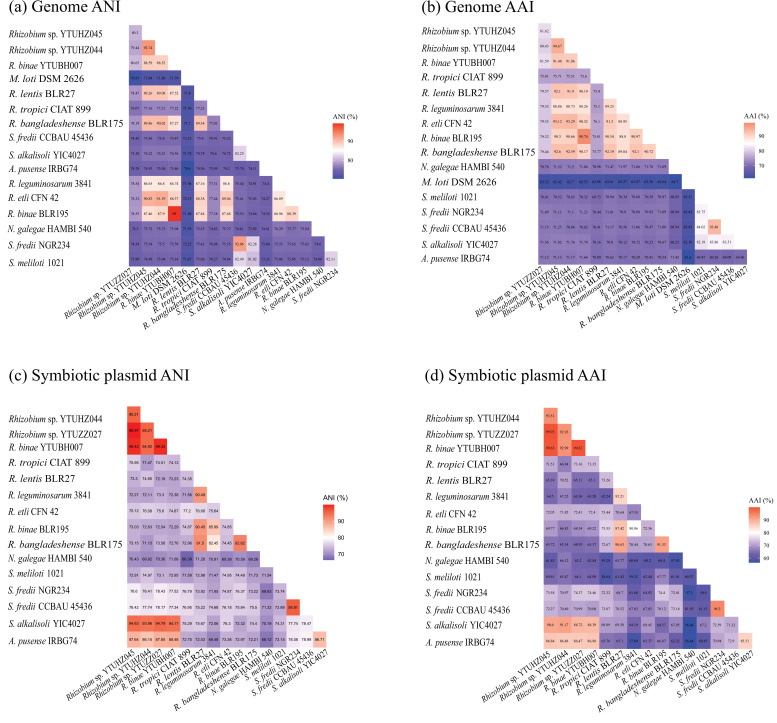
The heatmap of ANI and AAI values between the *S. cannabina*-nodulating rhizobia and the reference strains. The ANI heatmap generated by the complete genome sequences (**a**) and symbiotic plasmid (**c**); the AAI heatmap generated by the complete genome (**b**) and symbiotic plasmids (**d**).

According to the phylogenetic tree reconstructed using the whole-genome sequences, the four strains sequenced in this study were clustered within *Rhizobium* (Fig. 2a). The ANI values were 99.0 % between *R. binae* YTUBH007 and the type strain *R. binae* BLR195^T^, and less than 95 % among the three strains representing the unnamed genospecies and the reference strains of defined species (Fig. 3). However, the phylogenetic tree reconstructed using the complete sequences of pSyms (Fig. 2b) showed different topology relationships with the complete genome sequences. All the pSyms of *S. cannabina-*nodulating rhizobia*,* including *Sinorhizobium alkalisoli* YIC4027, *A. pusense* IRBG74 and the four *Rhizobium* strains sequenced in this study, formed a cluster sharing ANI values between 86.15 and 99.97 % (Fig. 2b). The highest ANI values occurred among *Rhizobium* sp. YTUHZ045, *Rhizobium* sp. YTUZZ027 and *R. binae* YTUBH007 (>99.42 %), which were consistent with their genome phylogenetic relationships (Fig. 2b). The AAI values showed the same tendencies as for ANI values (Fig. 3).

**Fig. 4.**
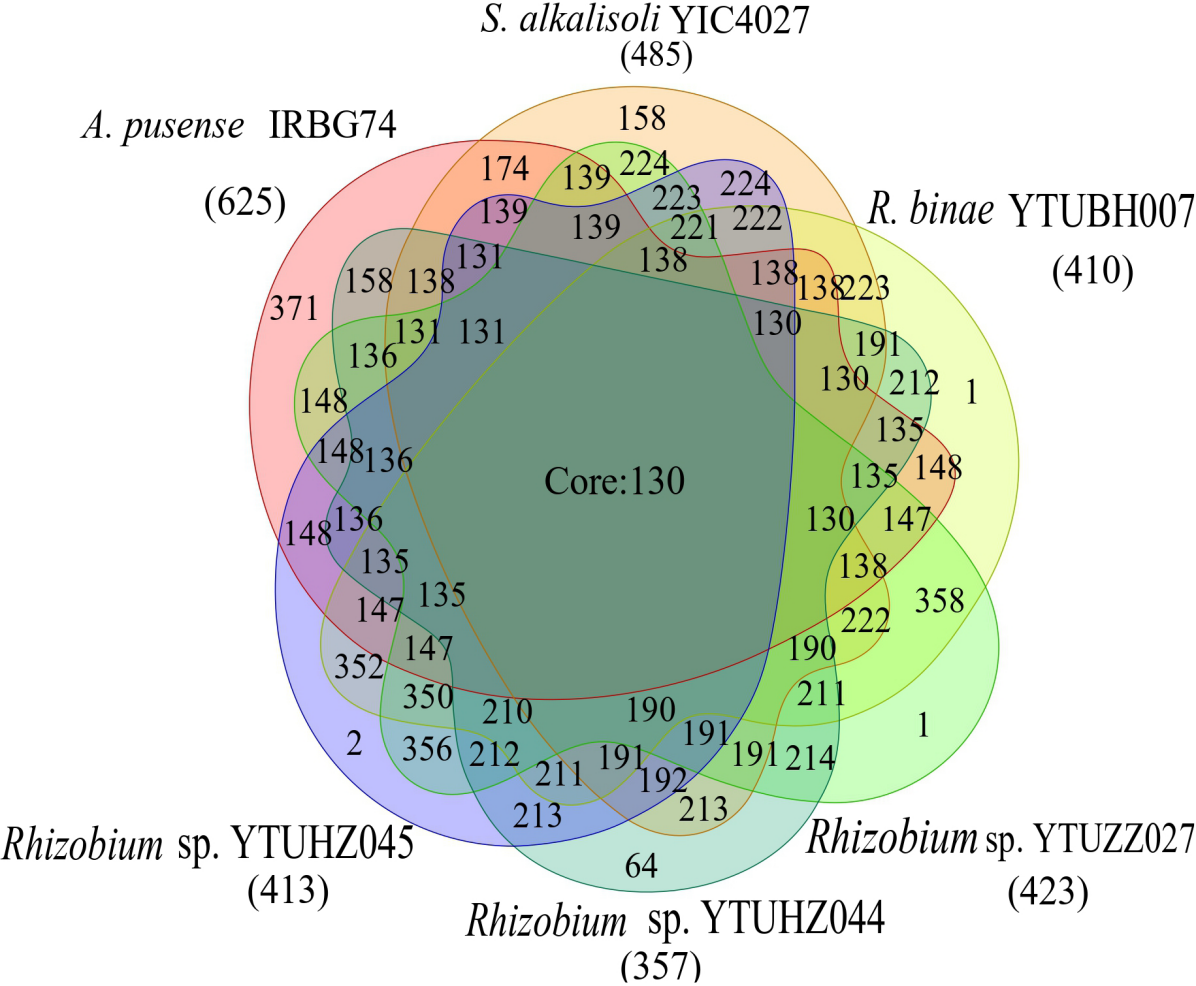
Homology analysis for symbiotic plasmids of *S. cannabina*-nodulating rhizobia. The diagram shows the number of common shared core genes, the numbers of genes shared by each two strains and the strain-specific genes for each strain.

A total of 130 core genes were determined among the six *S. cannabina*-nodulating rhizobial strains (Fig. 4), including 16 *nod*, 15 *nif* and 10 transposition genes (Table S4). In addition, another 82 common genes were shared by the four *S. cannabina*-nodulating *Rhizobium* strains (Fig. 4, Table S4), including 17 genes related to the T3SS and 17 genes related to conjugal transfer (Table S4), which were absent in *A. pusense* IRBG74. For strain-specific genes, *A. pusense* IRBG74 presented the highest number (371), followed by *Sinorhizobium alkalisoli* YIC4027 (158); while only one (* R. binae* YTUBH007 and *Rhizobium* sp. YTUZZ027) or two (* Rhizobium* sp. YTUHZ045) were detected for the *Rhizobium* strains. In addition, the common shared genes between each pair of the *Rhizobium* pSyms indicating that YTUBH007, YTUZZ027 and YTUHZ045 have closer relationships than that with YTUHZ044 (Fig. 4), consistent with the phylogenetic relationships and ANI values (Figs 2b and 3).

### Genetic organization of symbiosis and conjugal transfer related genes

**Fig. 5.**
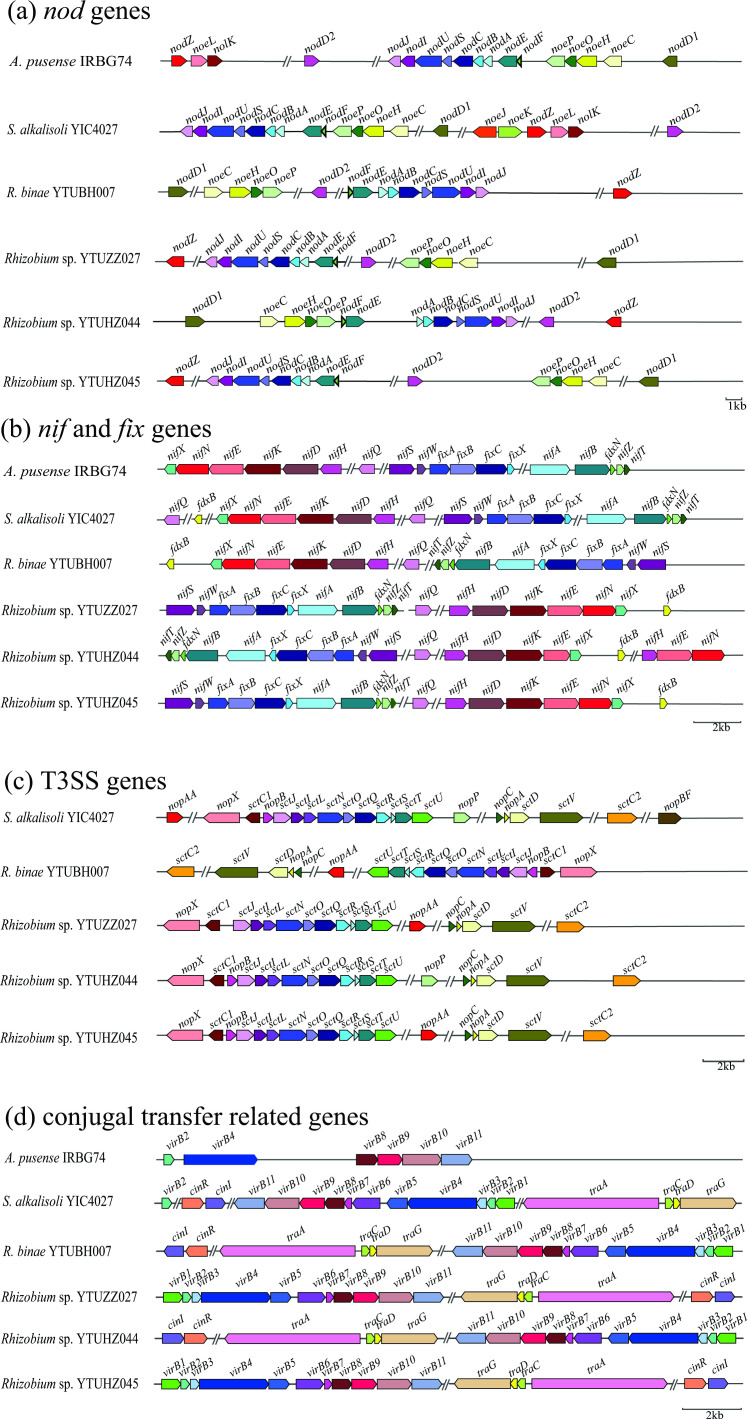
Genetic organization of symbiotic nitrogen fixation and conjugal transfer related genes in the symbiotic plasmid. (a) Nod factor synthesis genes; (b) nitrogen-fixation-related genes; (c) T3SS genes; (d) conjugal transfer related genes.

The plus direction of the pSym sequences was set the same as the *repABC* sequence. The organization and architecture of *nod*, *nif*, *fix*, T3SS and conjugation-related genes on pSyms were generated (Fig. 5). All the six *S. cannabina*-nodulating strains contain 16 *nod* genes, including *nodA*, *nodB*, *nodC*, *nodD1*, *nodD2*, *nodE*, *nodF*, *nodI*, *nodJ*, *nodS*, *nodU*, *nodZ*, *noeC*, *noeH*, *noeO* and *noeP* (Fig. 5a). However, *Sinorhizobium alkalisoli* YIC4027 and *A. pusense* IRBG74 possess another two genes, *noeL* and *nolK*; and the *four Rhizobium* strains contain genes *noeJ* and *noeK*. The nodulation genes were mainly distributed in two clusters: *nodABCSUIJ* and *noeCHOP*. Interestingly, *nodFE* clustered with *noeCHOP* in *A. pusense* IRBG74, *R. binae* YTUBH007, *Rhizobium* sp. YTUZZ027 and *Rhizobium* sp. YTUHZ045, and with *nodABCSUIJ* in *Sinorhizobium alkalisoli* YIC4027 and *Rhizobium* sp. YTUHZ044 (Fig. 5a).

For nitrogen-fixation-related genes, all the six strains contain 15 *nif* and *fix* genes, including *nifS*, *nifQ*, *nifN*, *nifK*, *nifH*, *nifE*, *nifD*, *nifB*, *nifA*, *nifX*, *nifW*, *fixA fixB*, *fixC* and *fixX* (Fig. 5b); while the four *Rhizobium* strains also harbour genes *fdxB* and *nifX*. Furthermore, two copies for both the genes *nifE* and *nifH* were detected in the pSym of *Rhizobium* sp. YTUHZ044. The *nif* genes were mainly distributed in three clusters: *nifABZT-fdxN*, *nifHDKENX* and *nifSW*; while the other *nif* genes are dispersed in the pSyms (Fig. 5b).

As for the T3SS-related genes, five of the *S. cannabina*-nodulating strains, excepting *A. pusense* IRBG74, contain the following 17 genes: *nopA*, *nopC*, *nopX*, *sctC1*, *sctC2*, *sctD*, *sctI*, *sctJ*, *sctL*, *sctN*, *sctO*, *sctQ*, *sctR*, *sctS*, *sctT*, *sctU* and *sctV* (Fig. 5c). No T3SS-related gene was detected in *A. pusense* IRBG74, while additional genes *nopBF* were found in *Sinorhizobium alkalisoli* YIC4027 and *nopP* in *Rhizobium* sp. YTUHZ044.

Similar to the situation for T3SS genes, no Dtr gene was found in *A. pusense* IRBG74, while complete Dtr gene sets including *cinI*, *cinR*, *traA*, *traC, traD* and *traG* were detected in the other five *S. cannabina*-nodulating strains. For Mpf genes, except for *virB1*, *virB3* and *virB5* being absent in the pSym of *A. pusense* IRBG74, all the other five *S. cannabina*-nodulating strains harboured *virB1* through *virB11*. Complete *oriT* sequences were predicted in pSyms of five *S. cannabina*-nodulating strains, except in *A. pusense* IRBG74, which did not harbour the *oriT* sequence (Fig. 5d **,** Table S5).

## Discussion

### Genome characteristics of *S. cannabina*-nodulating *Rhizobium* spp.

In our previous study, the *S. cannabina*-nodulating rhizobia across the world were classified as 16 species in four genera, and the symbiosis genes of all the strains formed a unique cluster [17]. Among them, the strains within different *Rhizobium* species presented extraordinarily highly conserved symbiosis genes [17], indicating that they might have acquired the symbiosis genes through HGT in a short evolutionary history. The symbiosis genes are usually located on pSyms [4, 37–39] or chromosomal symbiotic islands [40–44]. As for other fast-growing rhizobia, the symbiosis genes of *S. cannabina-*nodulating rhizobia *A. pusense* IRBG74 and *Sinorhizobium alkalisoli* YIC4027 were also located in a pSym [24, 45]. In the present study, we firstly evidenced the existence of pSym in the *S. cannabina*-nodulating *Rhizobium* species. According to the phylogenetic relationships (Fig. 2a) and the ANI values (Fig. 3), the four *Rhizobium* strains were classified as different species with one validly published (* R. binae *) and three new candidate species, which supported the previously reported species affiliation based upon MLSA (multilocus sequence analysis) [17, 18].

A single symbiosis plasmid was identified in each strain and both the genes *nodABC* and *nifHDK* essential for Nod factor synthesis and nitrogenase are located in the pSym, similar to the previously described pSyms [46]. The assembled pSyms in this study presented sizes of 345 to 402 kb and G+C content of 58.43–59.09 mol%, which was obviously lower than that of the chromosome 59.65–61.24 mol%, and was consistent with other *Rhizobium* strains [47]. The sizes of the detected pSyms were much smaller than those of the previously published *S. cannabina*-nodulating rhizobia *A. pusense* IRBG74 (585 kb) and *Sinorhizobium alkalisoli* YIC4027 (456 kb).

### Relationships among the pSyms of *S. cannabina*-nodulating rhizobia

The ANI and AAI values of the pSyms were consistent with the corresponding phylogenetic relationships based on the pSym sequences (Figs 2b and 3) and on the symbiosis genes [17, 18], indicating that the pSyms of *S. cannabina*-nodulating rhizobia have the same origin. Due to the greatly high identities, fewer strain-specific genes and more core genes among the pSyms in the four tested *Rhizobium* strains (Figs 3 and 4), it could be estimated that these strains acquired the plasmid in a short evolutionary history. Among the four *Rhizobium* strains, YTUHZ044 has the smallest pSym with the most strain-specific genes, supporting it having a more distant relationship with the other three *Rhizobium* strains (Fig. 4). Among the 130 core genes present in all the six pSyms, ten genes were related to transposition, which is consistent with the high abundance of insertion sequences in pSyms [48]. Other types of core genes in the pSyms of tested *Rhizobium* strains were those related to T3SS and conjugal transfer genes (Dtr- and Mpf-encoding genes), which were absent in *A. pusense* (Fig. 5c, d, Table S4). No identical pSyms were found among the tested strains, indicating that the pSyms might have been modified by the acceptor rhizobia after the HGT.

### Symbiotic characteristics of the pSyms

The nodulation process between legume and rhizobia is primarily incited by the Nod factor. In this study, the common *nod* genes were very similar among the six *S*. *cannabina*-nodulating strains, and the Nod factors they produce may have a complicated chemical structure modified by fucosylation, *N*-methylation, carbamoylation and arabinosylation, catabolized by NodZ, NodS, NodU and NeoP [49]. Thus, all the *Sesbania* rhizobia may secrete very similar decorated Nod factors, and the chemical structures may be strongly selected by the host, since *S. cannabina* only nodulated with rhizobia harbouring similar symbiosis genes [17, 50].

All the six *Sesbania*-nodulating rhizobia contained 15 complete *nif* and *fix* genes (Fig. 5b), including the *nif* core genes *nifHDK* encoding the full nitrogenase complex [51] and other nitrogen-fixation-related genes such as: *nifB*, which is needed for synthesis of the iron-molybdenum cofactor [52]; *nifW* and *nifZ*, whose products may be required for protection of the nitrogenase from oxygen [53]; the genes for the regulatory protein NifA [52]; the ferredoxin gene *fdxN*, which is essential for nitrogen fixation in *Sinorhizobium meliloti* [52]; *nifU* and *nifS* involved in the formation of the mature Fe-S cluster required for nitrogenase complex function [54]. However, *nifV* involved in synthesis of the iron-molybdenum cofactor [55] was absent in all the six strains; so, this cofactor might be provided by the host during symbiotic nitrogen fixation [56] in *S. cannabina*.

The T3SS widely distributed in rhizobial genomes determines the symbiotic efficiency, it can be completely necessary for nodule formation or can block nodulation during the rhizobium–legume symbiosis process [57]. It is a protein complex and secretes effector proteins from the cytosol of the bacteria into the host cell through a tube spanning the bacterial and host membranes, and it consists of needle structure proteins and effector proteins secreted by the needle [58]. In this study, except for *A. pusense* IRBG74, which is without any related genes, the other five strains contain genes encoding 14 structural genes: *sctQ*, *sctL*, *sctO* and *sctN* encoding proteins that form the cytoplasmic complex in the bacteria; *sctR*, *sctS*, *sctT*, *sctU* and *sctV* encoding proteins that form the export apparatus; *sctC1*, *sctC2*, *sctD* and *sctJ* encoding proteins of the basal body part; *sctI* encoding proteins of the needle part [58]. In addition, *nopA* encodes a key component of the extracellular part of the secretion machinery, whereas *nopX* encodes a part of the translocon that directs effector proteins across the plant plasma membrane [59, 60]. The only common effector protein shared by the five strains was NopC, which positively affects the symbiosis of *Sinorhizobium fredii* HH103 with soybean but negatively regulates its symbiosis with *Lotus japonicus* [61, 62]. Interestingly, *A. pusense* IRBG74 was successful for nodulation [63, 64] without any T3SS-related gene (Fig. 5), indicating that T3SS is unnecessary for *S. cannabina* nodulation.

### Transfer ability prediction for pSyms

For rhizobia, the symbiosis varieties (symbiovars) and effectiveness (nitrogen fixation) were mainly determined by the symbiosis genes located on pSyms or symbiotic islands [65]. However, the symbiosis genes could be transferred through transfer of the entire plasmid [4] or symbiotic island by integrative and conjugative elements (ICEs) to the receptor strain [4, 6, 7]. Comparison between the phylogenetic relationships, ANI values and AAI values of the whole genome and pSym (Table 1), and the Venn diagram (Fig. 4), clearly evidenced that the pSyms in the tested *S. cannabina-*nodulating rhizobia were acquired through plasmid transfer.

**Table 1.** Genome information for *S. cannabina*-nodulating *Rhizobium* spp

Strain/replicon	Feature	* Rhizobium binae * YTUBH007	* Rhizobium* sp. YTUHZ045	* Rhizobium* sp. YTUHZ044	* Rhizobium* sp. YTUZZ027
Genome	Total base pairs	6 641 388	6 745 774	6 550 840	6 610 703
	G+C content (mol%)	61.24	60.95	61.16	59.65
Chromosome	Length (bp)	4 494 605	4 505 147	4 665 784	3 803 027
	G+C content (mol%)	61.57	61.13	61.28	60.02
Plasmid 1	Length (bp)	521 020	1 064 782	550 430	2 049 752
	G+C content (mol%)	61.17	60.39	61.15	59.36
Plasmid 2	Length (bp)	492 537	503 456	517 681	402 479
	G+C content (mol%)	62.11	61.65	61.88	59.09
Plasmid 3	Length (bp)	394 444	396 883	345 554	355 445
	G+C content (mol%)	59.06	59.09	58.43	58.08
Plasmid 4	Length (bp)	302 945	275 506	263 799	–
	G+C content (mol%)	61.14	61.51	61.23	–
Plasmid 5	Length (bp)	225 588	–	207 592	–
	G+C content (mol%)	58.59	–	61.24	–
Plasmid 6	Length (bp)	109 298	–	–	–
	G+C content (mol%)	59.6	–	–	–
Plasmid7	Length (bp)	100 951	–	–	–
	G+C content (mol%)	59.09	–	–	–
Symbiotic plasmid	–	Plasmid 3	Plasmid 3	Plasmid 3	Plasmid 2

The transfer of pSym is mainly through conjugative transfer [4], and genes encoding Dtr and Mpf and the *oriT* sequence region are necessary for successful conjugative transfer [10, 36]. The *oriT* sequence is recognized by Dtr and then the transfer is elicited [14, 36]. The Dtr usually comprises necessary *tra* genes (*traACDG*) [12] and the regulatory genes *cinIR* or *traIR* [15]. *traA* encodes relaxase that interacts with *oriT* [12]; *traC* encodes ATPase, which is involved in pilus assembly and contributes to conjugation at the cell–cell contact stage [66, 67]; *traD* encodes the coupling protein TraD, a hexameric ring ATPase that forms the cytoplasmic face of the conjugative pore [11]; and *traG* encodes a large transfer protein, which consists of an inner membrane domain and a large periplasmic domain involved in pilus tip assembly and mating pair stabilization [11, 66]. Except for the *A. pusense* IRBG74 without any Dtr genes, the other five tested strains harboured *traA*, *traCDG* and the regulatory genes *cinI* and *cinR* (Fig. 5d), indicating they encompass the necessary *tra* genes.

The Mpf genes in rhizobial pSyms have two types, the complete *trb*-like systems such as that in *Sinorhizobium* sp. NGR234 [68], and *virB* T4SSs such as that in *Rhizobium etli* CFN42 and *Sinorhizobium meliloti* 1021 [12, 52]. The *virB* T4SS is usually composed of 12 genes (*virB1–11*/*D4*): *virB4* and *virB11* encode ATPases that coordinate the recruitment and processing of substrates, catalyse structural changes in the T4SS channel necessary for substrate passage and also regulate pilus biogenesis [13, 14, 69]; *virB3*, *virB6* and *virB8* encode integral inner membrane (IM) subunits that presumptively form an IM channel [14]; *virB1* encodes a transglycosylase that contributes to assembly of the channel across the murein layer [14]; and *virB7*, *virB9* and *virB10* encode outer membrane (OM)-associated subunits that form a structural scaffold for the portion of the channel spanning the periplasm and OM [70]. Except for *A. pusense* IRBG74 without *virB1*, *virB3* and *virB5*, the other five *Sesbania*-nodulating strains harboured a complete set of *virB* genes (Fig. 5d). However, *virD4* was absent in all the pSyms of *Sesbania*-nodulating rhizobia; furthermore, this gene was also absent in the self-transferrable pSyms in *Sinorhizobium meliloti* 1021 [71] and *R. etli* CFN42 [72] (data not shown). Thus, the transfer of pSyms in the *Sesbania*-nodulating rhizobia may be via a *virD*-independent way or by an unidentified gene with the same function.

Without *oriT*, *tra* and incomplete *vir* genes, the pSym of strain *A. pusense* IRBG74 seemed not self-transferable. However, the other five tested strains had the necessary genes of conjugal transfer including *oriT*, *tra* and the complete *virB*, indicating they could be transferred as the donor strain. The existence of diverse *S. cannabina*-nodulating rhizobia indicates their transferrable pSym could be transferred frequently, and the receptor strains adapted to the local conditions could establish symbiosis with *S. cannabina*. In this case, the receptor strains might be native bacteria without symbiosis genes [42] or could have an original pSym corresponding to other hosts. In the latter case, the original pSym may have been lost due to the incompatibility of two similar pSyms [73], and finally resulted in the receptor strain becoming the *S. cannabina*-specific rhizobium, such as *R. binae* YTUBH007. It is interesting to note *A. pusense* IRBG74 is the only *Agrobacterium* strain with symbiotic nitrogen fixation ability according to the genome sequences (578 *Agrobacterium* genomes from GenBank, data not shown), which was presumed to be loss of Ti plasmid and acquisition of the symbiotic plasmid from rhizobium [25, 64]. However, according to our study, the symbiotic plasmid of *A. pusense* IRBG74 is not self-transferrable, which is not consistent with any other species sharing the same symbiotic gene type with it [17]. Thus, we speculate the transfer-related element of the plasmid may have been lost in the evolutionary history of this species.

### Conclusion

Four *S. cannabina*-nodulating *Rhizobium* strains were sequenced and assembled at the replicon level and compared with another two related strains in this study. Each of the six strains harboured a pSym with size (345 554–402 479 bp) and genomic organization different from each other. The phylogenetic relationships, ANI and AAI values, and the shared genes among the pSyms indicated that they acquired the entire plasmid with a short evolutionary history. The *nod*, *nif*, T3SS and conjugal transfer related genes were located on the pSyms. All the *S. cannabina-*nodulating rhizobia presented very similar *nod* gene clusters, indicating that the legume rigorously selected the chemical structure of the Nod factor of the rhizobia; however, the T3SS might be unnecessary for the symbiosis with *S. cannabina*. The transferrable pSym of each strain contained *oriT*, *cinIR*, *traACDG* and complete *virB1–11* genes but without *virD*, indicating that they may be transferred through a *virD*-independent mode or by another unidentified gene.

## Supplementary Data

Supplementary material 1
