# Supplementary material for: Comparative genome analysis of Sesbania cannabina-nodulating Rhizobium spp. revealing the symbiotic and transferrable characteristics of symbiosis plasmids
Source: Microb Genom. 2023 May 3;9(5):mgen001004. doi: 10.1099/mgen.0.001004 (PMC10272884; doi:10.1099/mgen.0.001004)
Supplement: Supplementary material 1 [file mgen-9-1004-s001.pdf]

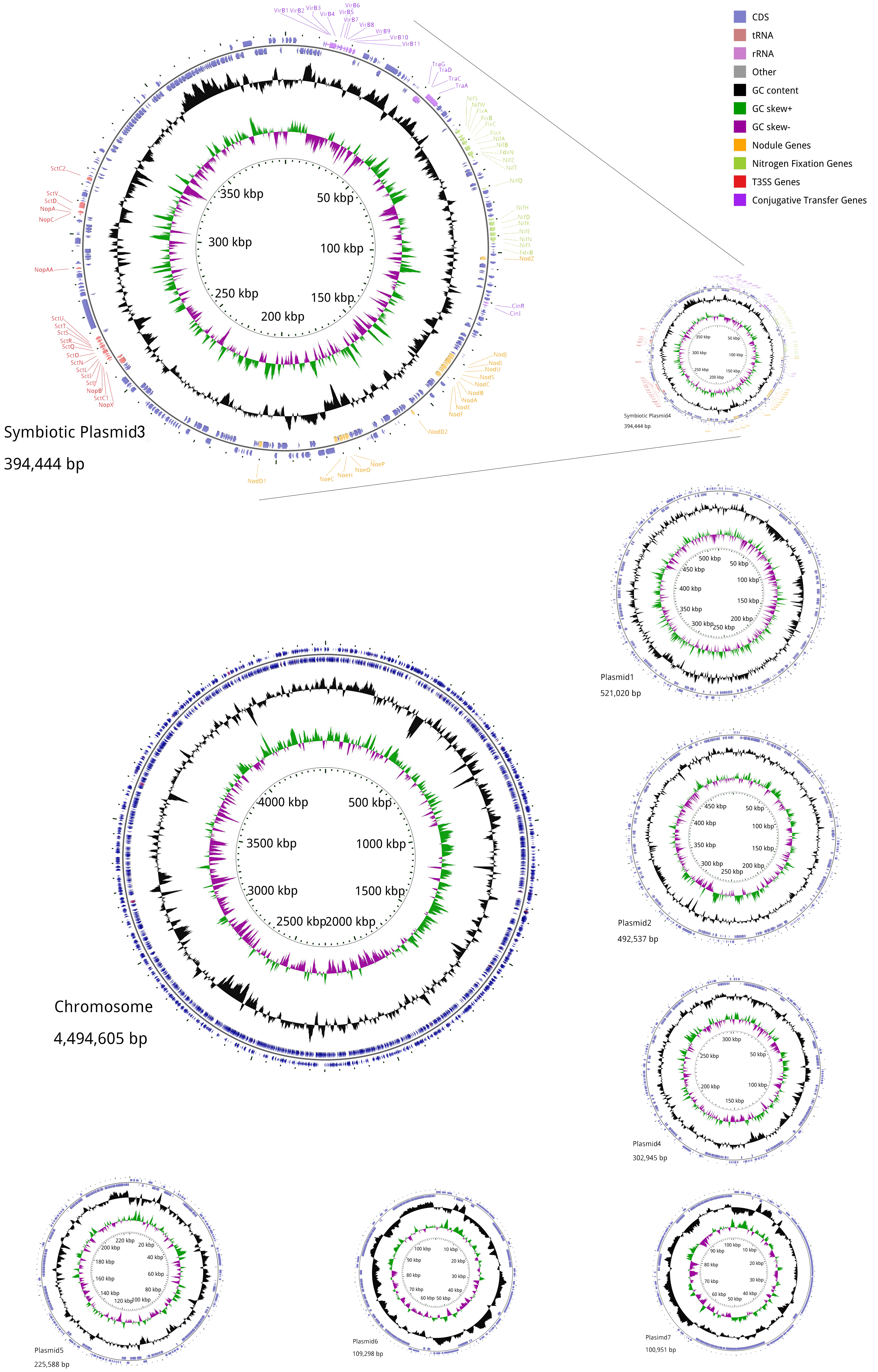

a: *Rhizobium binae* YTUBH007

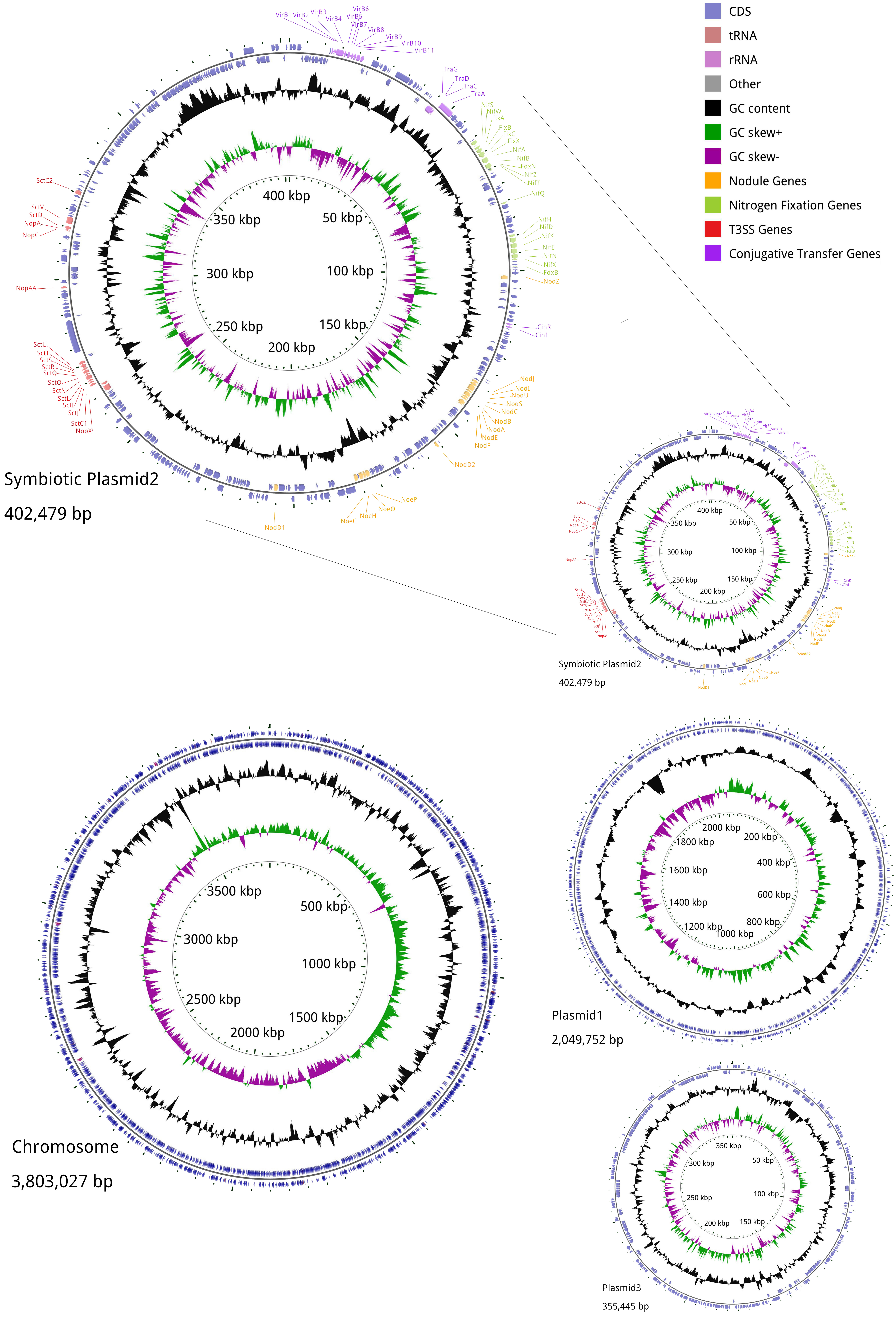

b: *Rhizobium* sp. YTUZZ027

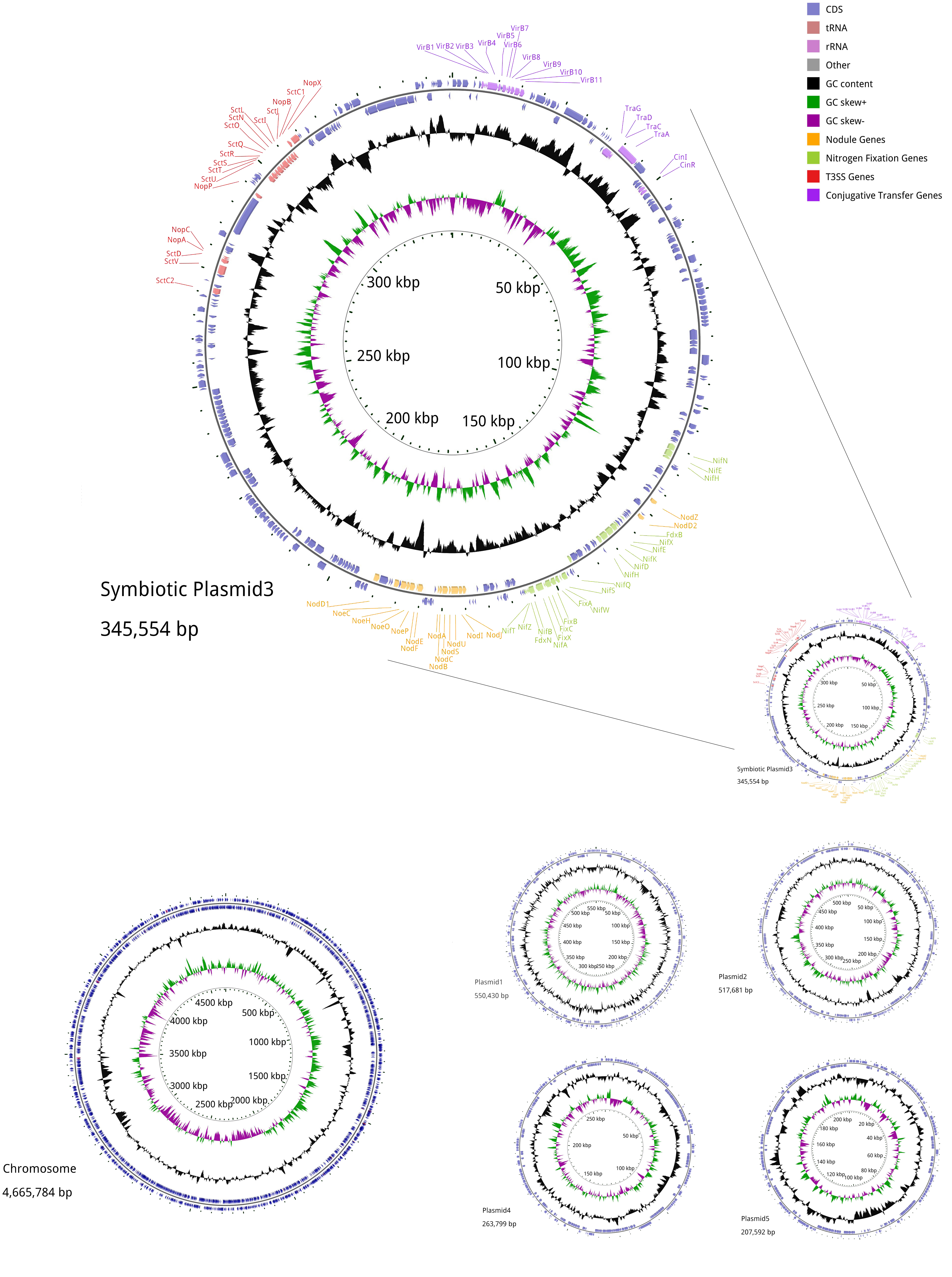

c: ***Rhizobium*** **sp. YTUHZ044**

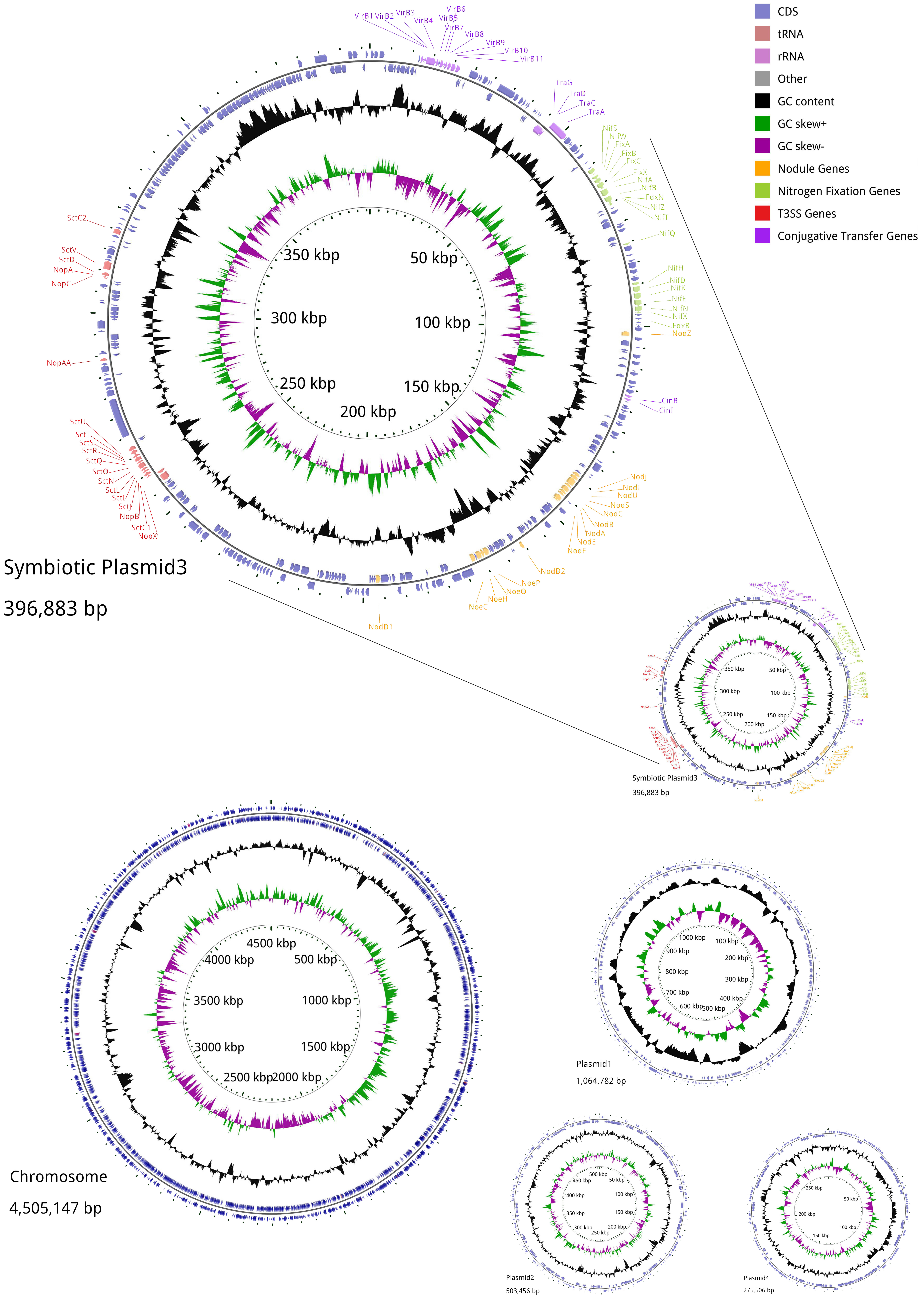

d: *Rhizobium* sp. YTUHZ045

Figure S1. Circular plots of the four *S. cannabina* nodualting *Rhizobium* spp. The outermost and second-outermost circles represent the predicted protein coding sequences on the forward and the reverse strand, respectively. Meanwhile, nodule genes are colored yellow, and nitrogen fixation genes are colored chartreuse, and T3SS genes are colored red, and conjugative transfer genes are colored magenta. The second-innermost and third-innermost circles show the GC skew values and GC content, respectively. GC skew values are colored purple for plus values and green for minus values. a: *Rhizobium binae* YTUBH007; b: *Rhizobium* sp. YTUZZ027; c: *Rhizobium* sp. YTUHZ044; d: *Rhizobium* sp. YTUHZ045.

Table S1 Genome information for *S. cannabina* nodulating rhizobia.

Strain/Replicon		*Rhizobium binae* YTUBH007	*Rhizobium* sp. YTUHZ045	*Rhizobium* sp. YTUHZ044	*Rhizobium* sp. YTUZZ027	*Agrobacterium pusense* IRBG74	*Ensifer alkalisoli* 4027
Genome	Total base pairs	6,641,388	6,745,774	6,550,840	6,610,703	5,464,982	6,128,433
	GC Content (%)	61.24	60.95	61.16	59.65	59.10	62.24
Chromosome	Length (bp)	4,494,605	4,505,147	4,665,784	3,803,027	2,844,565	3,690,234
	GC Content (%)	61.57	61.13	61.28	60.02	59.29	62.57
Plasmid 1	Length (bp)	521,020	1,064,782	550,430	2,049,752	2,035,452	1,981,775
	GC Content (%)	61.17	60.39	61.15	59.36	59.28	62.28
Plasmid2	Length (bp)	492,537	503,456	517,681	402,479	584,965	456,424
	GC Content (%)	62.11	61.65	61.88	59.09	57.48	59.34
Plasmid3	Length (bp)	394,444	396,883	345,554	355,445		
	GC Content (%)	59.06	59.09	58.43	58.08		
Plasmid4	Length (bp)	302,945	275,506	263,799			
	GC Content (%)	61.14	61.51	61.23			
Plasmid5	Length (bp)	225,588		207,592			
	GC Content (%)	58.59		61.24			
Plasmid6	Length (bp)	109,298					
	GC Content (%)	59.6					
Plasmid7	Length (bp)	100,951					

	GC Content (%)	59.09					
Symbiotic plasmid		Plasmid 3	Plasmid 3	Plasmid 3	Plasmid 2	Plasmid 2	Plasmid2

Table S2 The COG categorization for symbiotic plasmids CDSs of *S. cannabina*-nodulating rhizobia

COG function categories	*R. binae* YTUBH007	*Rhizobium* sp. YTUZZ027	*Rhizobium* sp. YTUHZ044	*Rhizobium* sp. YTUHZ045	*A. pusense* IRBG74	*S. alkalisoli* YIC4027	Total CDSs	Ratio
Energy production and conversion (C)	41	41	32	41	51	31	237	10%
Cell cycle control, cell division, chromosome partitioning (D)	4	4	6	4	4	4	26	1%
Amino acid transport and metabolism (E)	31	31	34	32	72	44	244	10%
Nucleotide transport and metabolism (F)	1	1	1	1	6	3	13	1%
Carbohydrate transport and metabolism (G)	8	8	8	8	47	22	101	4%
Coenzyme transport and metabolism (H)	5	5	3	5	7	17	42	2%
Lipid transport and metabolism (I)	10	10	7	10	25	14	76	3%
Translation, ribosomal structure and biogenesis (J)	4	4	0	4	3	0	15	1%
Transcription (K)	29	29	27	29	69	36	219	9%
Replication, recombination and repair (L)	100	105	60	100	52	85	502	21%
Cell wall/membrane/envelope biogenesis (M)	4	4	4	4	16	13	45	2%
Cell motility (N)	8	8	8	8	3	8	43	2%
Posttranslational modification, protein turnover, chaperones (O)	13	13	4	13	14	7	64	3%

Inorganic ion transport and metabolism (P)	9	9	8	9	31	16	82	3%
Secondary metabolites biosynthesis, transport, and catabolism (Q)	12	12	12	12	28	15	91	4%
Function unknown (S)	55	55	51	53	111	80	405	17%
Signal transduction mechanisms (T)	14	14	9	14	22	11	84	3%
Intracellular trafficking, secretion, and vesicular transport (U)	22	22	22	21	10	29	126	5%
Defense mechanisms (V)	5	5	4	5	3	4	26	1%
Total	375	380	300	373	574	439	2441	

Table S3 KEGG Pathway classification for symbiotic plasmids CDSs of *S. cannabina*-nodulating rhizobia

	A. pusense IRBG74	*S. alkalisoli* YIC4027	*R. binae* YTUBH007	*Rhizobium* sp. YTUZZ027	*Rhizobium* sp. YTUHZ045	*Rhizobium* sp. YTUHZ044	Ratio
Carbohydrate metabolism	55	34	21	21	21	20	15%
Energy metabolism	19	8	9	9	9	8	5%
Lipid metabolism	7	4	3	3	3	2	2%
Nucleotide metabolism	1	1	1	1	1	3	1%
Amino acid metabolism	46	31	27	27	27	25	16%
Metabolism of other amino acids	9	5	6	6	6	4	3%
Metabolism of cofactors and vitamins	12	11	6	6	6	8	4%
Biosynthesis of other secondary metabolites	9	7	4	4	4	3	3%
Xenobiotics biodegradation and metabolism	39	28	24	24	24	34	15%
Translation	1		1	1	1		0%
Folding, sorting and degradation	2	1	1	1	1	1	1%
Membrane transport	37	33	30	30	30	32	17%
Signal transduction	9	8	7	7	7	3	4%
Cellular community - prokaryotes	15	12	12	12	12	13	7%
Cell motility	3	1	2	2	2	2	1%
Aging	2	1	1	1	1	1	1%

Cancer: overview	4	4	4	4	4	3	2%
Cancer: specific types	1	1	1	1	1	1	1%
Infectious disease: bacterial	4	2	2	2	2	2	1%
Cardiovascular disease	2	2	1	1	1	1	1%
Drug resistance: antimicrobia	5		1	1	1		1%
Drug resistance: antineoplastic	1	1	1	1	1	1	1%
Glycan biosynthesis and metabolism	2	5				1	1%
Metabolism of terpenoids and polyketides	5						0%
Replication and repair	3						0%
Transport and catabolism	1						0%
Endocrine and metabolic disease	2	2					0%
Total	296	202	165	165	165	168	

Table S4 The core symbiotic plasmid genes categorization of *S. cannabina*-nodulating rhizobia

No.	Protein	Function
1	-	Hypothetical protein
2	MsmX	ATPases associated with a variety of cellular activities
3	-	Aldehyde dehydrogenase family
4	-	Component of the pyruvate dehydrogenase (PDH) complex
5	-	Oxidoreductase NAD-binding domain
6	-	PFAM MOSC domain
7	Msi124	Aminotransferase class I and II
8	DapAch3	Belongs to the DapA family
9	-	L-lactate permease
10	-	Belongs to the GST superfamily
11	-	Helix_turn_helix ASNC type
12	-	Phosphotransferase enzyme family
13	-	Gamma-aminobutyric acid catabolic process
14	-	Homoserine dehydrogenase
15	IciA	Transcriptional regulator
16	SugE	Small multidrug resistance protein
17	-	Catalyzes the formation of L-homocysteine from O- succinyl-L-homoserine (OSHS) and hydrogen sulfide
18	-	transport
19	-	Binding-protein-dependent transport system inner membrane component
20	-	ABC-type amino acid transport signal transduction systems, periplasmic component domain
21	-	ABC-type histidine transport system, ATPase component
22	-	PFAM B3 4 domain protein
23	-	Helix-turn-helix XRE-family like proteins
24	-	Sigma-54 interaction domain
25	-	Hydrolase, alpha beta fold family
26	KgtP	Permeases of the major facilitator superfamily
27	AcsA	Catalyzes the conversion of acetate into acetyl-CoA (AcCoA)
28	McpA3	Chemotaxis protein
29	-	Transcriptional regulator, AraC family
30	-	Cobalamin synthesis protein cobW C-terminal domain
31	-	Protein of unknown function (DUF1097)
32	-	Nitrile hydratase, alpha chain
33	-	Nitrile hydratase beta subunit

34	-	Transcriptional regulator
35	AmaB	Peptidase family M28
36	GlnQ	ABC transporter
37	-	Hypothetical protein
38	-	Creatinase/Prolidase N-terminal domain
39	NodD1	Nodulation protein D1 (NodD1)
40	-	Acyltransferase family
41	-	Transcription regulator
42	-	Transposase
43	-	Belongs to the 'phage' integrase family
44	NoeC	Nodulation protein C (NoeC)
45	-	GtrA-like protein
46	NoeH	Nodulation protein H (NoeH)
47	NoeO	Nodulation protein O (NoeO)
48	NoeP	Nodulation protein P (NoeP)
49	-	Integrase core domain
50	-	Transposase and inactivated derivatives
51	-	Helix-turn-helix domain
52	-	Hypothetical protein
53	NodD2	Nodulation protein D2 (NodD2)
54	-	Transcriptional regulator
55	-	Transposase
56	-	Transposase
57	NodF	Nodulation protein F (NodF)
58	NodE	Nodulation protein E (NodE)
59	NodA	Nodulation protein A (NodA)
60	NodB	Nodulation protein B (NodB)
61	NodC	Nodulation protein C (NodC)
62	NodS	Nodulation protein S (NodS)
63	NodU	Nodulation protein U (NodU)
64	NodJ	Nodulation protein J (NodJ)
65	-	Uncharacterised protein family
66	HisC3	Class-II pyridoxal-phosphate-dependent aminotransferase family
67	-	Luciferase-like monooxygenase
68	ISPkr1	Transposase
69	-	Transposase and inactivated derivatives
70	-	Hypothetical protein
71	-	Transposase, IS3 IS911 family protein
72	-	Winged helix-turn helix
73	-	Location bacterial cytoplasm
74	-	Transposase and inactivated
75	NodZ	Nodulation protein Z (NodZ)
76	-	Transposase

77	-	Nitrogen fixation
78	NifX	Nitrogen fixation protein X （NifX）
79	NifN	Nitrogen fixation protein N （NifN）
80	NifE	Nitrogen fixation protein E （NifE）
81	NifK	Nitrogen fixation protein K （NifK）
82	NifD	Nitrogen fixation protein D （NifD）
83	NifH	Nitrogen fixation protein H （NifH）
84	-	Luciferase-like monooxygenase
85	-	Enoyl-(Acyl carrier protein) reductase
86	-	SnoaL-like domain
87	-	Cytochrome P450
88	DctA	Responsible for the transport of dicarboxylates
89	NifQ	Nitrogen fixation protein Q （NifQ）
90	-	Peroxiredoxin
91	FixU	Nitrogen fixation protein FixU
92	NifZ	Nitrogen fixation protein Z （NifZ）
93	FdxN	4Fe-4S binding domain
94	NifB	Nitrogen fixation protein B （NifB）
95	NifA	Nitrogen fixation protein A （NifA）
96	FixX	Could be a 3Fe-4S cluster-containing protein
97	FixC	FAD dependent oxidoreductase
98	FixB	Electron transfer flavoprotein FAD-binding domain
99	FixA	Electron transfer flavoprotein domain
100	NifW	Nitrogen fixation protein W （NifW）
101	NifS	Nitrogen fixation protein S （NifS）
102	IscA	Belongs to the HesB IscA family
103	-	Hypothetical protein
104	-	Protein of unknown function (DUF736)
105	-	Proposed nucleic acid binding domain
106	-	Belongs to the bacterial histone-like protein family
107	-	Protein of unknown function (DUF2493)
108	-	Toprim domain
109	-	C-terminal domain on Strawberry notch homologue
110	-	Hypothetical protein
111	-	Hypothetical protein
112	-	Hypothetical protein
113	-	ParB-like nuclease domain
114	VirB11	Type IV secretion system proteins VirB11
115	VirB10	Type IV secretion system proteins VirB10
116	VirB9	Type IV secretion system proteins VirB9
117	VirB8	Type IV secretion system proteins VirB8
118	VirB4	Type IV secretion system proteins VirB4

119	VirB2	Type IV secretion system proteins VirB2
120	RepC	Replication protein C (RepC)
121	RepB	Replication protein B (RepB)
122	RepA	Replication protein A (RepA)
123	-	NmrA-like family
124	-	HxlR-like helix-turn-helix
125	-	FCD
126	-	Belongs to the LDH2 MDH2 oxidoreductase family
127	-	Alcohol dehydrogenase
128	-	Aldehyde dehydrogenase family
129	-	hydratase
130	-	Belongs to the binding-protein-dependent transport system permease family
131	MegL	Cys/Met metabolism PLP-dependent enzyme
132	AspA	Aspartate ammonia-lyase
133	DapA	Catalyzes the condensation of (S)-aspartate-beta- semialdehyde (S)-ASA and pyruvate to 4-hydroxy- tetrahydrodipicolinate (HTPA)
134	MegL	Cystathionine beta-lyases cystathionine gamma-synthases
135	AzoR	Catalyzes the reductive cleavage of azo bond in aromatic azo compounds to the corresponding amines.
136	AzoR	Oxidoreductase activity, acting on other Nitrogenous compounds as donors
137	-	IS66 Orf2 like protein
138	NodI	Nodulation protein I (NodI)
139	-	Hypothetical protein
140	-	Hypothetical protein
141	CinI	Acyl-homoserine-lactone synthase
142	-	Transmembrane secretion effector
143	FdxB	4Fe-4S dicluster domain
144	-	Hypothetical protein
145	-	Hypothetical protein
146	-	Evidence 4 Homologs of previously reported genes of
147	-	Hypothetical protein
148	TraA	Conjugal transfer protein TraA
149	TraC	Conjugal transfer protein TraC
150	TraD	Conjugal transfer protein TraD
151	TraG	Conjugal transfer protein TraG
152	-	SpoVT AbrB
153	-	Protein of unknown function (DUF1173)

154	-	Antitoxin of toxin-antitoxin stability system
155	-	ParD-like antitoxin of type II bacterial toxin-antitoxin system
156	VirB5	Type IV secretion system proteins VirB5
157	VirB3	Type IV secretion system proteins VirB3
158	VirB1	Type IV secretion system proteins VirB1
159	-	ProQ/FINO family
160	-	N-6 DNA Methylase
161	-	ABC-type branched-chain amino acid transport systems, periplasmic component
162	LivG1	ABC-type branched-chain amino acid transport systems ATPase component
163	-	Phasin protein
164	ISPbe1	PFAM transposase IS3 IS911 family protein
165	-	Hypothetical protein
166	-	tail collar domain protein
167	MrlI	Autoinducer synthase
168	OmpR	Transcriptional regulatory protein
169	PazS	pseudoazurin
170	-	Hypothetical protein
171	-	Pilus biogenesis CpaD protein
172	SctC2	Type III secretion system protein SctC2
173	QseB	Transcriptional regulatory protein
174	-	Hypothetical protein
175	-	FOG TPR repeat
176	SctV	Type III secretion system protein SctV
177	SctD	Type III secretion system protein SctD
178	NopA	Type III secretion system protein NopA
179	NopC	Type III secretion system protein NopC
180	-	Shikimate kinase
181	SctU	Type III secretion system protein SctU
182	SctT	Type III secretion system protein SctT
183	SctS	Type III secretion system protein SctS
184	SctR	Type III secretion system protein SctR
185	SctQ	Type III secretion system protein SctQ
186	SctO	Type III secretion system protein SctO
187	SctN	Type III secretion system protein SctN
188	SctL	Type III secretion system protein SctL
189	SctI	Type III secretion system protein SctI
190	SctJ	Type III secretion system protein SctJ
191	SctC1	Type III secretion system protein SctC1
192	NolX	Type III secretion system protein NolX

193	-	Nitrile hydratase beta subunit
194	GlnQ	ATPases associated with a variety of cellular activities
195	YahB	LysR substrate binding domain
196	-	chemotaxis protein
197	ISUnCu14	Transposase
198	-	Hypothetical protein
199	-	Hypothetical protein
200	-	Hypothetical protein
201	-	Hypothetical protein
202	RhiA	Protein RhiA
203	CinR	Transcriptional regulator CinR
204	ISRel26	Winged helix-turn helix
205	VirB7	Type IV secretion system proteins VirB7
206	-	Hypothetical protein
207	LivH	Belongs to the binding-protein-dependent transport system permease family
208	-	Integrase core domain
209	-	Zeta toxin
210	-	Zeta toxin
211	VirB6	Type IV secretion system proteins VirB6
212	YciC	Cobalamin synthesis protein cobW C-terminal domain

Note: 1-130 were the core genes present in the symbiotic plasmid of the six *S. cannabina* nodulating rhizobia, and 131-212 were the additional common shared genes by four *Rhizobium* spp. sequenced in this study. C: Energy production and conversion; D: Cell cycle control, cell division, chromosome partitioning; E: Amino acid transport and metabolism; G: Carbohydrate transport and metabolism; H: Coenzyme transport and metabolism; I: Lipid transport and metabolism; K: Transcription; L: Replication, recombination and repair; M: Cell wall/membrane/envelope biogenesis; N: Cell motility; O: Posttranslational modification, protein turnover,chaperones; P: Inorganic ion transport and metabolism; Q: Secondary metabolites biosynthesis, transport and catabolism; S: Function unknown; T: Signal transduction mechanisms; U: Intracellular trafficking, secretion, and vesicular transport; V: Defense mechanisms.

Table S5 The *oriT* sequence of the symbiotic plasmid of the *S. cannabina*-nodulating rhizobia.

Strains	The predicted *oriT* sequence
S. alkalisoli YIC4027	AGGGCGCAATATACGTCGCTGGCGCGACGTGCTTG
A. pusense IRBG74	Not detected
R. binae YTUBH007	AGGGCGCAATATACGTCGCTGGCGCGACGTGCTTG
Rhizobium sp. YTUZZ027	AGGGCGCAATATACGTCGCTGGCGCGACGTGCTTG
Rhizobium sp. YTUHZ044	AGGGCGCAATATACGTCGCTGGCGCGACGTGCTTG
Rhizobium sp. YTUHZ045	AGGGCGCAATATACGTCGCTGGCGCGACGTGCTTG
